# Desmoid Tumor of the Popliteal Fossa during Pregnancy

**DOI:** 10.1155/2015/262654

**Published:** 2015-02-05

**Authors:** Wolfram Weschenfelder, Robert Lindner, Christian Spiegel, Gunther Olaf Hofmann, Matthias Vogt

**Affiliations:** Department of Trauma, Hand and Reconstructive Surgery, University Hospital Jena, Erlanger Allee 101, 07747 Jena, Germany

## Abstract

Desmoid tumors are fibroblastic neoplasms that have an intermediate behavior with a highly aggressive infiltrative growth arising from deep muscle or aponeurosis. We present the case of a 34-year-old woman that developed a painless mass in the right popliteal fossa during pregnancy after intracytoplasmic sperm injection and hormonal therapy. The MRI scan showed a hyperintense mass of 6,7 cm × 4,7 cm × 3,8 cm surrounding the lateral head of the gastrocnemius muscle. The open biopsy was done one week after delivery, and the histology showed a desmoid tumor. We performed the resection one week later and found the common peroneal nerve completely surrounded by the tumor. The close resection due to the neurolysis was the reason why an adjuvant radiation with 56 Gy was done. The last clinical examination, 18 month later, did not show any signs of recurrence and an excellent functional outcome. This case demonstrates the possible influences of pregnancy and hormonal therapy on the evolution of desmoid tumors.

## 1. Introduction

The desmoid tumor is a fibroblastic one also called “deep” or “aggressive” fibromatosis that arises from deep muscle or aponeurosis. It is classified by the WHO as a fibroblastic tumor of intermediate (locally aggressive) behavior with an infiltrative growth and a high tendency for local recurrence but no known metastasis [1, 2]. It is a rare disease with an incidence of about 2–4 per million with a slight female predominance [3]. It is associated with familial adenomatous polyposis (FAP) and Gardner's syndrome. Other known risk factors are prior surgery, trauma, oral contraceptives, pregnancy, and reproductive age. The histopathological examination reveals uniform spindle cells in a dense collagen stoma with only few mitosis and only rare areas of necrosis [1, 4–7].

In this paper, we report the case of a desmoid tumor developing during pregnancy in the right popliteal fossa, compromising the bifurcation of the sciatic nerve.

## 2. Case Presentation

A 34-year-old woman, gravida 1, para 2, noticed a swelling and indolent mass at the back of the right lower leg and popliteal fossa during the 5th month of pregnancy. She had undergone five attempts of intracytoplasmic sperm injection (ICSI) and hormonal therapy previous to this pregnancy. The gestation had been without pathological findings to this point and the family history of the woman did not show any malignant tumors. The first medical consultation due to the swelling was in the 6th month at her general practitioner, who performed a sonography and advised an MRI scan. This was finally done at the 11th july 2012 which showed a hyperintense mass of 6,7 cm × 4,7 cm × 3,8 cm surrounding the lateral head of the gastrocnemius muscle. The X-ray of the knee was without any pathological findings.

**Figure 1:**
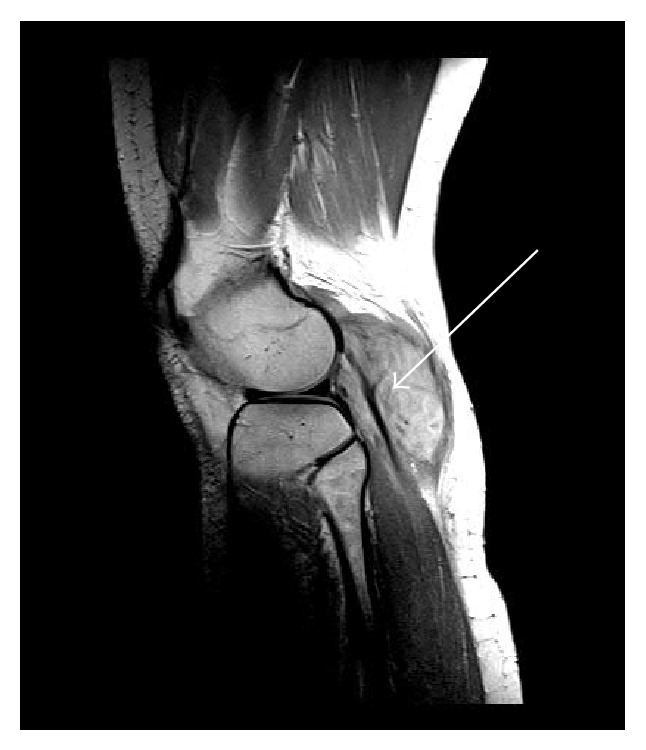
MRI scan, sagittal view.

**Figure 2:**
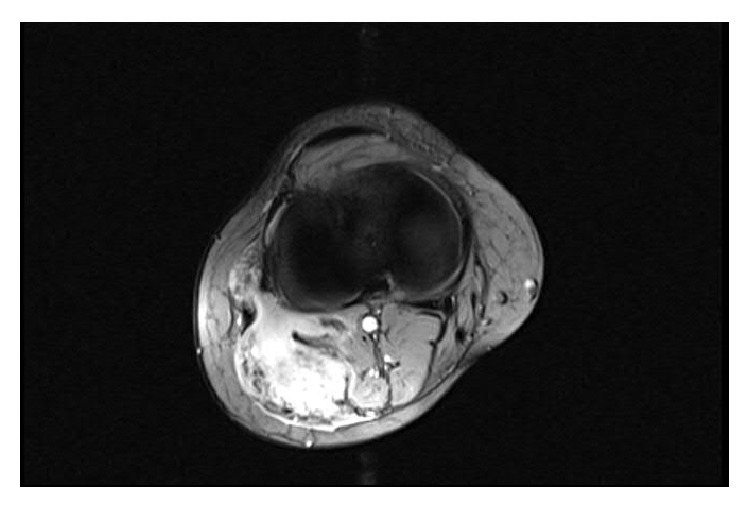
MRI scan, axial view.

The first presentation in our clinic for musculoskeletal tumors was 7 weeks before the calculated delivery date. The clinical examination showed a firm mass at the lateral back of the right lower leg, 10 cm in length from the popliteal fossa to distal (see Figures 1 and 2) adherent to the underlying muscles. The flexion of the knee was limited to 100°. The peripheral blood circulation and sensibility were intact at the day of presentation, but the patient described electric paresthesias occurring from time to time radiating from the knee along the lateral leg to the foot.

**Figure 3:**
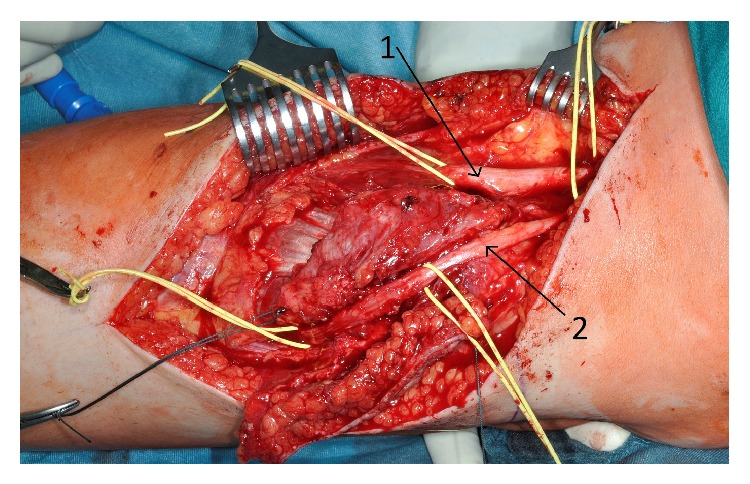
Intraoperative picture: 1 tibial nerve, 2 common peroneal nerves.

**Figure 4:**
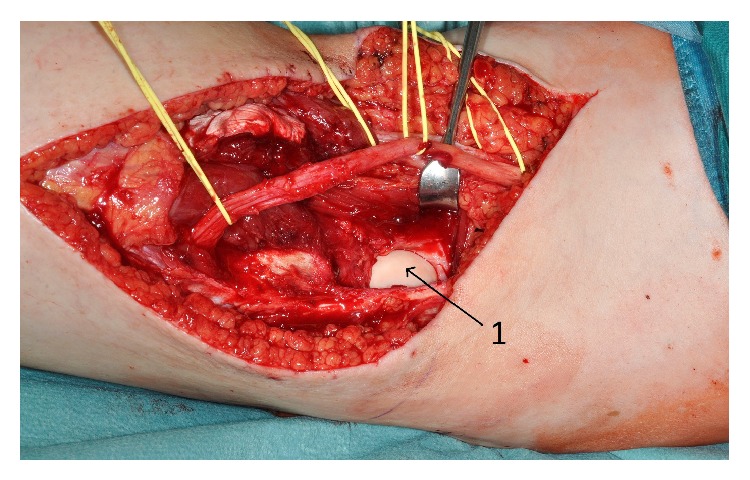
Intraoperative picture: 1 dorsal aspect of condylus lateralis.

**Figure 5:**
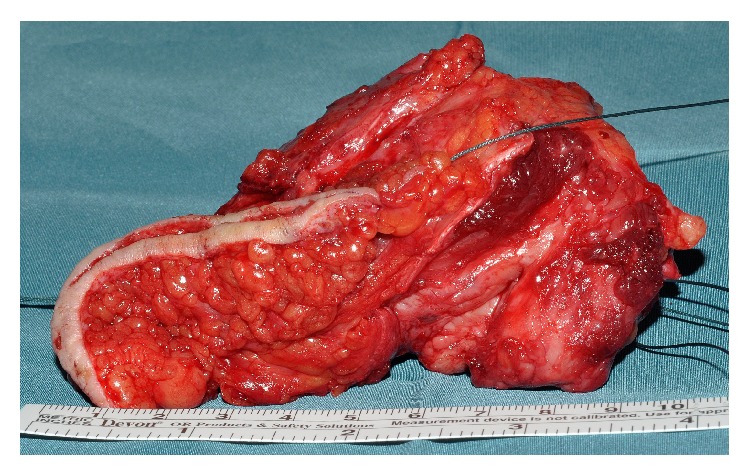
Resected tumor including skin and scar of biopsy.

**Figure 6:**
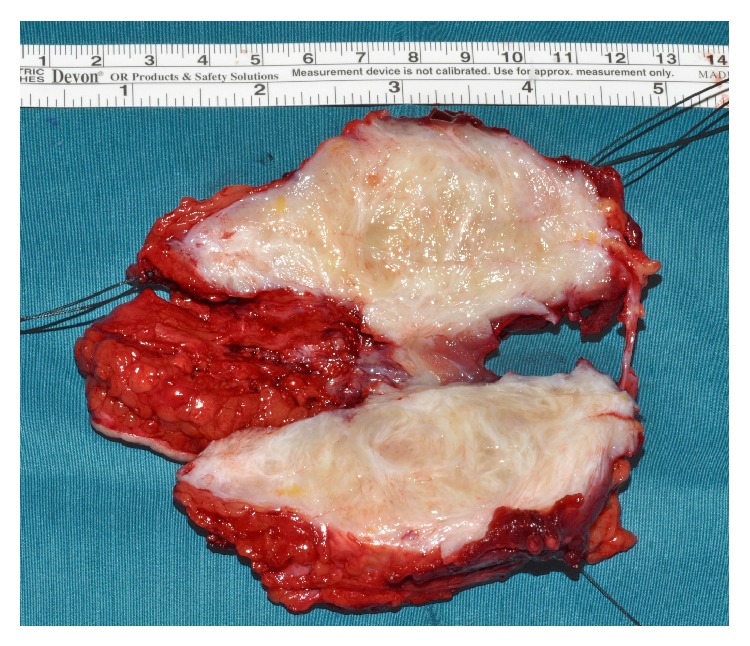
Resected tumor cut longitudinal.

The results of anamneses and clinical examination and MRI diagnostic and the differential diagnosis were discussed with the patient and further treatment was planned. We recommended an open biopsy due to the close anatomic relation to the tibial and common peroneal nerve which could cause complications within a fine needle biopsy. We decided to observe the tumor and perform the open biopsy one week after the delivery (which was a secondary caesarean section) at the 09th August 2012. The biopsy was done in a face-down position using a posterior approach to the knee. We found the communal peroneal nerve surrounded by the superficial portion of the desmoid tumor and it was mobilized to get a representative biopsy of 1 cm³. The nerve was marked with a vessel loop in preparation of the total excision that was scheduled for one week later. The histopathological findings proved the tumor to be a desmoid one and we performed the complete resection on the 16th of August 2012. The tumor was situated on the surface of the lateral head of the gastrocnemius muscle, infiltrating the top of the head of the fibula and the lateral parts of the dorsal capsule of the knee. The communal peroneal nerve was surrounded by tumor 6 cm in length and had to be dissected, even though the tibial nerve was displaced by the desmoid tumor (see Figures 3–6). The microscopy of the desmoid tumor showed R0 resection with a tumor-free margin of minimal one millimeter and proved the initial diagnosis of an extra-abdominal desmoids fibromatosis. The case was then presented in our multidisciplinary tumor board and a postoperative radiation was recommended to reduce the risk of recurrence especially in the area of the epineural neurolized common peroneal nerve. This was done from the 23rd of October until the 29th of November 2012 with a total radiation level of 56 Gy.

The routine follow-ups did not show any pathological abnormalities. The last clinical examination on the 7th of July 2014 showed an indolent scar after posterior approach to the right lower leg and popliteal fossa without palpable mass or skin defects. The movement of the knee had no limitations (extension/flexion 0/0/130°) and the patient described no restrictions of walking. The MRI scan during the follow-ups did not show any signs of recurrence. The patient achieved a Knee Society Score of 95 points indicating an excellent postoperative function.

## 3. Discussion

This case shows that swelling or indurations of an extremity during pregnancy might be a sign for a tumor that need further diagnostics and treatment. The localization of desmoid tumors is generally classified as intra-abdominal in the abdominal wall or extra-abdominal. Even though pregnancy-associated desmoids typically occur in the abdominal wall, there are few cases of extra-abdominal growth during gestation [1]. Our case was complicated by a very unfavorable position, with direct displacement of the bifurcation of the sciatic nerve in the tibial nerve and common peroneal nerve and immuring of it. This manifested itself by beginning neurological symptoms and restriction of movement of the knee. This corresponds to the typical clinical signs of desmoids such as initially painless mass with increasing restrictions on the movement of adjacent joints. However, this initial behavior is also shown by malignant soft tissue sarcomas. The locally aggressive growth, reminiscent of the behavior of sarcomas, was confirmed intraoperatively by signs of infiltration of the surrounding soft tissue, in particular the dorsal knee joint capsule and the head of the fibula.

Although the first description of a desmoid tumor goes back to MacFarlane in 1832 and Müller named the disease desmoid tumor in 1838, the exact pathology is not yet fully understood. The various etiological factors of desmoid fibromatosis, such as prior surgery, trauma, FAP, and Gardner's syndrome, have already been mentioned. In our case, however, the most likely effect seems to be due to sex hormones. It is generally accepted that women of reproductive age have an increased risk for the development of desmoid tumors especially during pregnancy [1, 2, 4, 5, 8, 9]. Our patient had received hormone therapy prior to the ICSI. An influence of the increased LH and FSH levels on tumor genesis of demoids have not been described in any publication yet.

The first presentation of a patient with a new soft tissue mass should always lead to further diagnostics after completion of anamnesis and clinical examination. These include sonography and for tumors of a size above 5 cm or subfascial growth a MRI scan for accurate assessment. The two purposes are to get the diagnosis if already possible just by MRI firstly and secondly to determine the next steps in particular regarding surgical resection. X-ray or CT diagnostics are only required if the MRI shows a possible erosion of the bone and a resection and subsequent reconstruction might be necessary. In our patient, we found a very close anatomic relationship to important nerve structures, so that the biopsy was carried out as an open biopsy by us.

In the treatment of desmoid tumors, new alternatives emerged especially in primary nonresectable locations in the last years. Some authors recommend, for clinically inconspicuous and stable desmoid tumors, regular check-ups and no specific treatment. However, due to the radiological not absolutely clear distinction to low-grade soft tissue sarcomas, we believe even these cases need histological confirmation of the diagnosis. Desmoid tumors with mass effects, neurological symptoms, or restricted movement range of a joint should be treated by surgical resection if possible, like in our case. Because of the high recurrence rates of 19–77%, adjuvant radiation therapy is a well-established option today and was used for our patient. The use of systemic therapies especially for nonresectable desmoid tumors has been increasing in the last years and includes cytotoxic agents, antiestrogen hormonal therapy (e.g., tamoxifen), and imatinib [1, 4, 10–12].

Known prognostic factors for recurrence after surgical excision are an extra-abdominal localization, a size of more than 7 cm, and an age under 37 years [3]. Our patient fulfilled all these risks, which is why we combined the wide surgical excision with adjuvant radiotherapy. The postoperative outcome is excellent regarding the function and at present time there is no sign of recurrence.

In conclusion, it must be emphasized that the desmoid tumor is a semimalignant soft tissue tumor which does not metastasize, but it unfortunately shows a very aggressive and infiltrative growth; therefore, it should not be underestimated. The standard treatment for symptomatic and growing desmoids is the surgical wide excision followed by adjuvant therapy. Particularly, in patients with a high risk of recurrence or relapse, the treatment with tamoxifen seems to be a promising option according to the recent studies.
